# “Vanishing Penis” and Urinary Retention due to Locally Destructive Penile Cancer

**DOI:** 10.1100/tsw.2009.14

**Published:** 2009-03-01

**Authors:** Petros Sountoulides, Athanasios Bantis, Ioannis Zachos, Irene Asouhidou, Athanasios Pantazakos

**Affiliations:** Department of Urology, Agios Andreas Regional Hospital, Patras, Greece

**Keywords:** penile cancer, urinary retention, vanishing penis

## Abstract

Penile carcinomas are relatively rare. They usually arise from precancerous lesions and present in the form of ulcerative or exophytic tumors. They rarely give rise to urinary symptoms and complications, and are usually easy to diagnose. We present a case of an 82-year-old man with chronic urinary retention due to urethral dissemination by a locally destructive penile lesion. The penis was literally “vanished” by the lesion down to the level of the pubic bone without, interestingly, having spread to the local lymph nodes or given rise to distant metastases. A temporary suprapubic catheter was placed, followed by a perineal urethrostomy in order to reverse the established renal failure.

## CASE

An 82-year-old patient was brought to our hospital by his relatives who, over the past few days, had noticed a gradual deterioration of his mental status, accompanied by low urine output, fatigue, and anorexia. According to his relatives, the patient was living alone and had been wearing pads for the last 6 months due to deteriorating urinary incontinence for which he never sought medical advice. The patient was under no medication whatsoever. History taking from the patient was tedious; however, he admitted noticing an exophytic, painless, penile lesion some months ago for which he, again, did not ask for medical advice.

Physical examination revealed marked suprapubic tenderness due to a largely distended urinary bladder. Examination of his external genitalia revealed complete absence of the shaft and glans of the penis, replaced by a hard, ulcerative lesion. In accordance with the patient's history, the finding was attributed to destruction of the penile shaft and glans by an invasive, ulcerated, obviously neglected, penile carcinoma (Fig. 1). Physical examination of regional lymphatics was negative. His prostate felt normal on DRE and his scrotal examination was insignificant. The urethral meatus was not visible and attempts at catheterization were fruitless because of local tumor dissemination and obstruction of the urethra from dense neoplastic tissue.

Blood results showed evidence of severe renal insufficiency (blood urea: 347 mg/dl; creatinine: 14.1 mg/dl; $K^+$: 5.5 mmol/l), while ultrasonography in the E.R. showed bilateral hydronephrosis and a largely distended bladder. A suprapubic catheter was placed under ultrasound guidance and immediately drained 1200 ml of urine (Figs. 2 and 3).

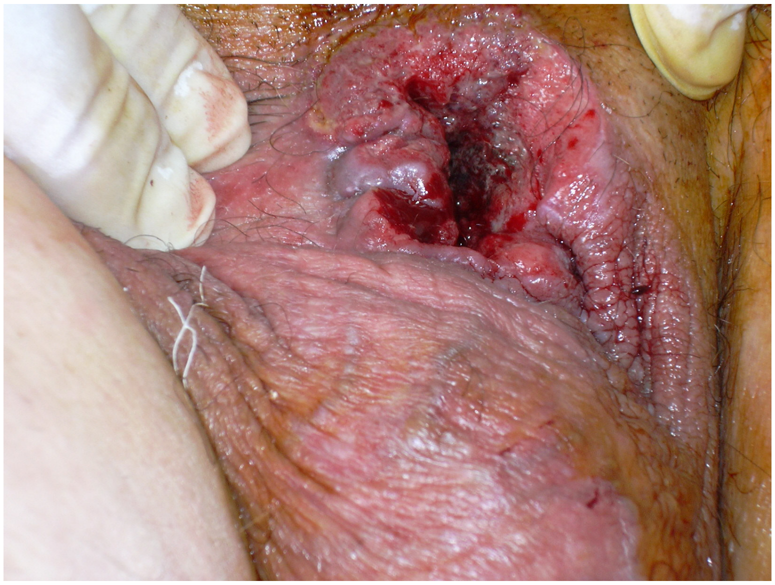

**FIGURE 1.** Ulcerative lesion in base of penis with urethral infiltration.

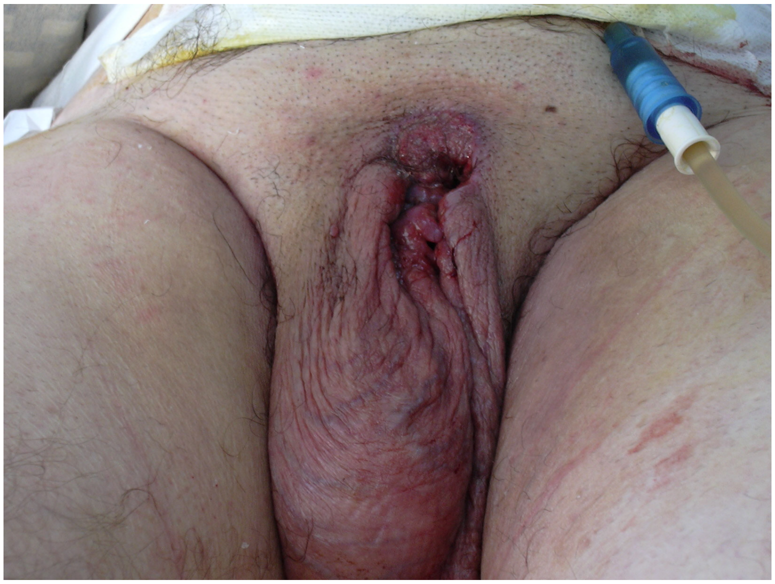

**FIGURE 2**. Suprapubic catheter placed due to unsuccessful catheterization of the urethra.

After establishment of normal renal function during the following days, the patient was subjected to a helical, contrast-enhanced CT of the pelvis and abdomen, which failed to detect pathologically enlarged lymph nodes or distant metastases.

Subsequently, the patient underwent extended surgical excision of the neoplastic tissue around the bulbar urethra at the level of the crus of the penis and a proximal perineal urethrostomy was developed.

The pathology report showed a grade II squamous cell carcinoma with central keratinization and negative surgical margins (Fig. 4). In the absence of distal metastases, we recommended that the patient receive adjuvant radiotherapy, but he decided against it. Subsequent follow-up CT scans and biannual physical examinations are normal 2 years later.

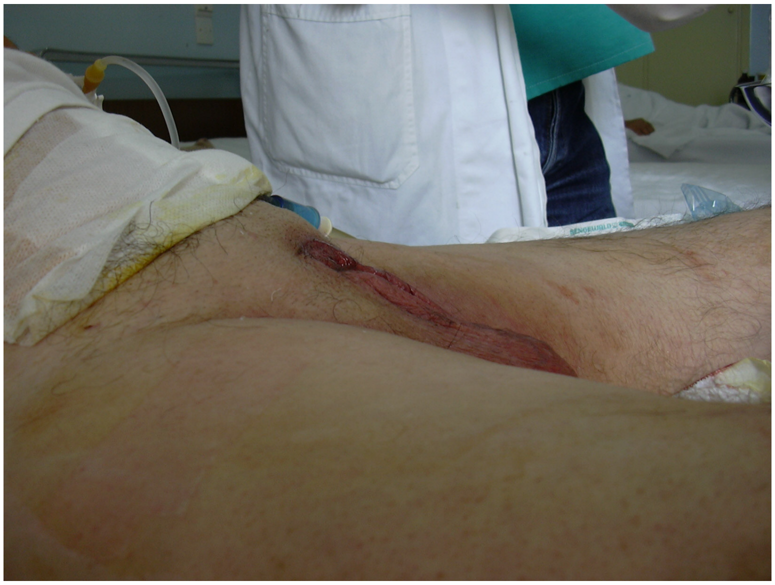

**FIGURE 3**. Profile view of “vanished” penis.

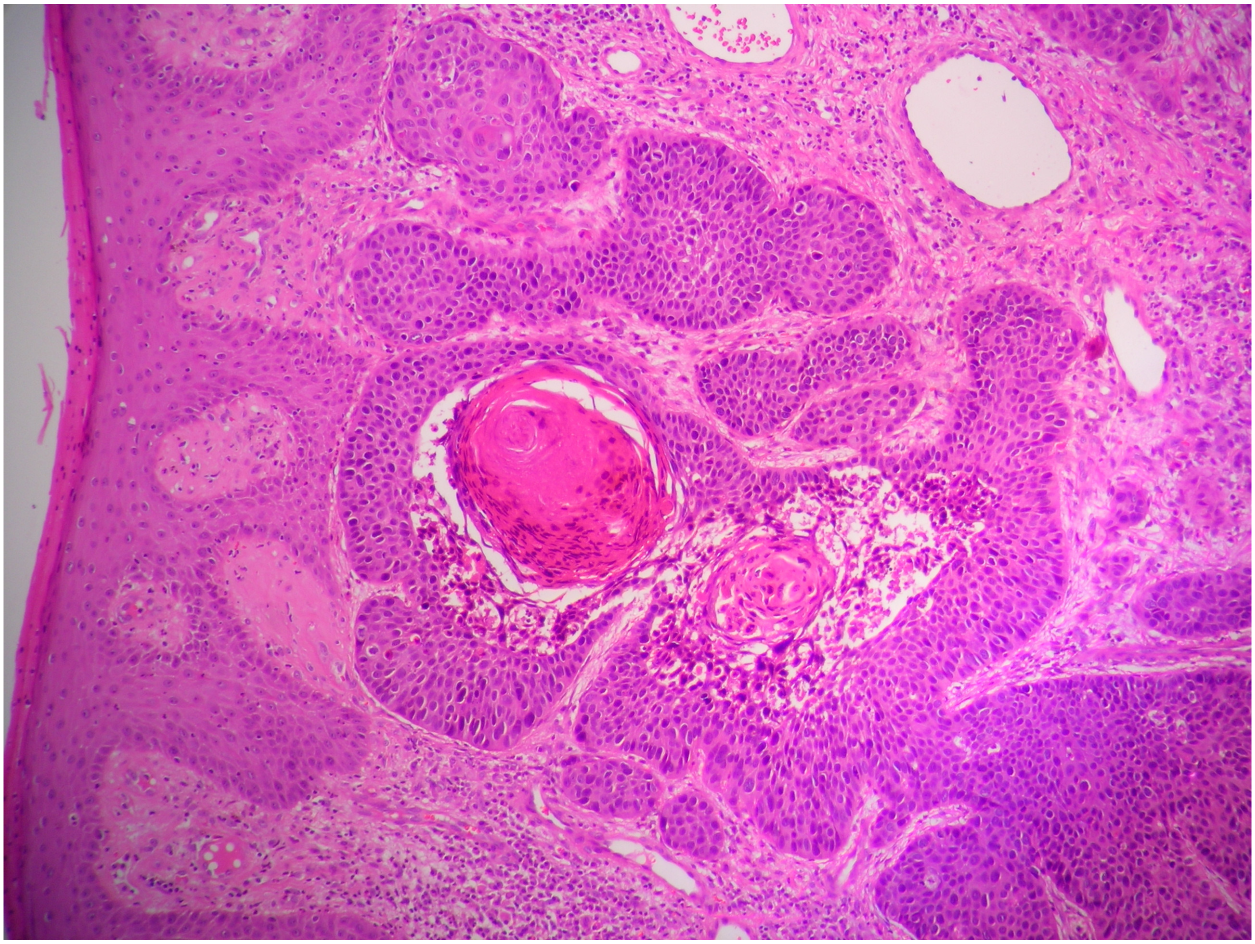

**FIGURE 4.** Pathology: In the dermis of penile mucosa, nests of neoplastic squamous cells with central keratinization (H&E, reduced from ×100).

## DISCUSSION

Penile cancer is a relatively uncommon malignancy accounting for only 0.4–0.6% of cancers diagnosed in Europe and the U.S. Penile cancer incidence rises at the age of 60 and peaks at around 80 years of age[1]. Squamous cell cancer represents the most common histologic subtype of penile cancer and accounts for 0.1–0.9 new cases per 100,000 males per year in the Western World[2].

If left untreated, penile cancer will rarely cause necrotic lesions with partial amputation of the shaft or urinary retention due to obstruction at the level of the urethra. To our knowledge, we report the first case of squamous cell carcinoma causing the penis to literally “vanish” due to extensive tissue destruction, with concomitant urinary retention caused by urethra infiltration.

There are only a few publications on urinary retention caused by penile cancer lesions and all of them refer to metastatic lesions of the penis, usually from prostate cancer[3,4]. In another case, urinary retention was accompanied by painful erection in a patient with primary squamous cell lung cancer and penile metastases[5].

Regarding the aspect of penile destruction, all of the cases reported in the literature share a common ischemic etiology caused by underlying vascular disease. The main characteristic in these cases is the element of severe pain caused by ischemic necrosis of the organ, which differs from our case where penile destruction was relatively painless. Penile ischemic necrosis causing organ loss has been reported as a result of Wegener’s granulomatosis[6], Buerger’s disease[7], and end-stage renal disease (ESRD)[8]. In these cases, penile necrosis resulted from ischemia due to occlusion or obliteration of the penile, internal pudendal, or iliac arteries. Patients with ESRD under long-term hemodialysis have a known a high incidence of vascular calcifications (VC), with penile artery calcifications visible in X-ray films in up to 19% of patients[9]. In these cases, penile necrosis is considered a consequence of ischemia caused by accelerated VC in ESRD patients[8].

Probably the most intriguing aspect of the case presented here is the absence of early or late lymph node involvement or distant metastases, in spite of the aggressive local progression of the tumor. According to a large retrospective study from Solsona et al., stage T2-3 and grade II penile cancers, as the one in our case, share an 83.3% risk of positive lymph nodes[10]. Interestingly, lymph nodes were negative on both physical examination and CT imaging. In this case, and according to the EAU guidelines[11], there is no reason to proceed with sentinel node biopsy of the inguinal nodes due to the high incidence of false-negative results[12].

Judging from the outcome of the patient after a 2-year follow-up, there is reason to believe that his TNM stage was actually N0, although there have been reports of cases with late lymph node involvement[13].

Finally, penile reconstruction following perineal urethrostomy was impossible in our case due to the extent of the penile destruction and the patient’s age. However, penile reconstruction following total or partial penectomy can be technically feasible, and is currently offered in specialized centers with acceptable cosmetic and functional results[14].

## References

1. Lynch, D.F., Jr. and Pettaway, A.C. (2002) Tumors of the penis. In *Campbell’s Urology*. 8th ed. Vol. 4. Walsh, P.C., Retik, A.B., Vaughan, E.D., and Wein, A.J., Eds. WB Saunders, Philadelphia.
2. Jemal, A., Siegel, R., Ward, E., Murray, T., Xu, J., and Thun, M.J. (2007) Cancer statistics, 2007. *CA Cancer J. Clin.* **57,** 43–66.
3. Bar-Moshe, O., Abdul-Sater, A., and Vandendris, M. (1991) Acute urinary retention secondary to cavernous metastases from a prostatic tumor. *Prog. Urol.* **1,** 1042–1045.
4. Spreen, S.A., Keys, R.H., Jr., and Evans, A.T. (1975) Acute urinary retention secondary to metastatic prostatic carcinoma to the penis: a case report. *J. Urol.* **113,** 59.
5. Yokoi, K., Miyazawa, N., Muraki, J., Nakazono, M., Imura, G., and Shinamura, K. (1992) Penile metastasis from lung cancer. *Jpn. J. Clin. Oncol*. **22,** 297–299.
6. Matsuda, S., Mitsukawa, S., Ishii, N., and Shirai, M. (1978) A case of Wegener's granulomatosis with necrosis of the penis. *Tohoku J. Exp. Med.* **118,** 145–151.
7. Orhan, I., Onur, R., Akpolat, N., and Semercioz, A. (2004) Penile amputation in Buerger's disease - an unusual case of organ loss. *Scand. J. Urol. Nephrol.* **38(2),** 188–189.
8. Gwel, S., Yaycioglu, O., Kilinc, F., Torun, D., Kyaselcuk, F., and Ozkardes, H. (2004) Penile necrosis in end-stage renal disease. *J. Androl.* **25(1),** 25–29.
9. Dalal, S., Gandhi, V.C., Yu, A.W., Bhate, D.V., Said, R.A., Rahman, M.A., and Ing, T.S. (1992) Penile calcification in maintenance hemodialysis patients. *Urology* **40(5),** 422–424.
10. Solsona, E., Iborra, I., Rubio, J., Casanova, J.L., Ricos, J.V., and Calabuig, C. (2001) Prospective validation of the association of local tumor stage and grade as a predictive factor for occult lymph node micrometastasis in patients with penile carcinoma and clinically negative inguinal lymph nodes. *J. Urol.* **165,** 1506–1509.
11. Solsona, E., Algaba, F., Horenblas, S., Pizzocaro, G., Windahl, T.; European Association of Urology (2004) EAU guidelines on penile cancer. *Eur. Urol.* **46(1),** 1–8.
12. Pettaway, C.A., Pisters, L.L., Dinney, C.P.N., Jularbal, F., Swanson, D.A., von Eschenbach, A.C., and Ayala, A. (1995) Sentinel lymph node dissection for penile carcinoma: the M.D. Anderson Cancer Center Experience. *J. Urol.* **154,** 1999–2003.
13. Ficarra, V., Zattoni, F., Artibani, W., Fandella, A., Martignoni, G., Novara, G., Galetti, T.P., Zambolin, T., Kattan, M.W.; G.U.O.N.E. Penile Cancer Project Members. (2006) Nomogram predictive of pathological inguinal lymph node involvement in patients with squamous cell carcinoma of the penis. *J. Urol.* **175(5),** 1700–1704.
14. Summerton, D.J., Campbell, A., Minhas, S., and Ralph, D.J. (2005) Reconstructive surgery in penile trauma and cancer. *Nat. Clin. Pract. Urol.* **2(8),** 391–397.
